# The bioinformatics of genetic origins: how identities become embedded in the tools and practices of bioinformatics

**DOI:** 10.1186/2195-7819-9-7

**Published:** 2013-09-13

**Authors:** Jan van Baren-Nawrocka

**Affiliations:** Department of Philosophy and Science Studies, Radboud University Nijmegen, Postvak 77, P.O. Box 9010, 6500, GL Nijmegen, The Netherlands

**Keywords:** Bioinformatics, Genomics, Life sciences, Computers, Software, Databases, Population identities, Naturalisation, Essentialisation

## Abstract

In the life sciences, where large data sets are increasingly setting the stage for research, the role of bioinformatics is expanding. This has far-reaching consequences, not only for the way research is done, but also for the way this research affects our understanding of human identity. Using two case studies of practices involving bioinformatics, the software program *Structure* and the *Genome of the Netherlands* project, I will argue that bioinformatics and its tools can be understood as ‘infrastructure’ as described by Bowker and Star. A number of value decisions are involved in the development of such tools. However, once the tools are ready for use, these values tend to blend into the background of the research. This may lead to the ‘naturalisation’ and ‘essentialisation’ of value-imbued aspects of population identities such as nationality, ethnicity and race.

## Introduction

Bioinformatics is a core discipline of the life sciences in general and of genomics in particular. Regardless of whether it is seen as ‘just’ a toolset or as a driving force for research, in view of the increasing complexity of the research of molecular connections, the role of bioinformatics as a technology and as a discipline is clearly intensifying.

As (Rose 2007) has argued, human life is increasingly regarded as being shaped at the molecular level. As a crucial discipline in the research of human functioning on a molecular level, bioinformatics not only affects the scientific disciplines involved but also how we define ourselves as human beings.

(Zwart 2009) has argued that the knowledge produced by the life sciences on the basis of bioinformatics, restructures our understanding of human identities on three levels: the collective or species level, the genealogical or historical level and the individual or personal level. In this paper, I will concentrate on the genealogical or historical level. Whereas Zwart focuses on content, i.e. changing knowledge structures and knowledge claims that inform and restructure identities, I will focus on the research *practices* by which this knowledge is produced, looking at how certain specific population identities are shaped or ‘enacted’^1^.

‘Population identities’ refers to those elements of individual identity that are based on membership of a group that lays claim to a shared origin.

In genetics discourses this shared origin is enacted as a genetic connection between humans and our ancestors, human as well as non-human. In population genetics, a genetic connection is made between current human inhabitants of certain locations, whose ancestors are presumed to have lived there as well (indigenous populations), and humans who might share this ancestry but live elsewhere. In this way the latter’s connection to this place is thus enacted as ‘origin’.

In this paper I will analyse the role of bioinformatics in the enactment of these origins. More specifically, I will argue that specific enactments of population identities as genetic categories are materially embedded in bioinformatics tools. When these tools become part of the infrastructure, they tend to blend into the background, including the values (in this case population identities) that they incorporate. This often results in the naturalisation of these incorporated values, that is, they are taken for granted and no longer questioned (Bowker and Star 2000). Naturalisation of population identities as genetic categories can lead to their essentialisation as natural phenomena, meaning that they are seen as a reality of nature (Epstein 2007; M’charek 2005).

I have selected two case studies in which bioinformatics as a research discipline plays a central role. In both cases, different forms of population identity are enacted as genetic in very different ways. In the first case study, the software program *Structure*, developed for determining population structures, was used to analyse the way assumptions about populations find their way into a software algorithm. Where this algorithm is used for determining population structure, it contributes to specific enactments of population identities, inadvertently revoking notions of ethnicity and race as biological phenomena. Subsequently, in the second case study, the *Genome of the Netherlands*, a database allegedly containing the genomic profile of the Dutch population, was used to analyse how bioinformatics and specifically this database, contributes to the enactment of nationality as a genetic category.

In the following, I will first outline my methods and key concepts. Next, I will describe and analyse the cases and their consequences one by one, connecting them to broader discourses on population identities. I will conclude the paper with tentative recommendations for keeping identities flexible in the context of genomics.

## Methods and concepts

### Methods

My research builds on important work that has been done on how identities are being enacted in (molecular) sciences internally (M’charek 2005) and in the interactions of sciences with society (Lipphardt and Niewöhner 2007; Epstein 2007). This research constitutes the backdrop of my analysis, in which I will tease out the specific role of bioinformatics and show how values become embedded in genomics infrastructures through the use of bioinformatics practices and bioinformatics tools.

To address this issue, I have made use of three sources of information. First, I took part in a number of activities where bioinformatics is practised: two courses in applied bioinformatics (as a student) and three bioinformatics conferences (as an attendant, one time also presenting a poster). While functioning mainly as a background for my analysis, this source of information has been vital for writing the paper. It shaped my view of, and familiarity with, bioinformatics in terms of the technical aspects of the field as well as current developments and discussions.

Second, I conducted a series of interviews^2^ with researchers working in the field of bioinformatics or in close collaboration with it. To be precise: six interviews were conducted with bioinformatics researchers, one with a population geneticist and one with a human geneticist. Rather than asking them directly about the role of bioinformatics in shaping identity, I asked the interviewees to describe what they do as a researcher, taking the interview from there.

Third, to substantiate the analysis, I analysed a selection of published materials such as scientific publications, project websites and published interviews related to the case studies.

### Concepts

Two central concepts deserve further explanation: ‘enactment’ and ‘naturalisation’.

I draw on Mol’s use of the concept of enactment to describe how objects and phenomena are shaped in and by the *practices* they are part of (Mol 2002). Consisting of daily events and activities, practices include language, discourse and actions as well as their material context (Mol 2002). Enactment includes all these factors as part of shaping reality. Mol used this concept because it allowed her to concentrate on practices as such, rather than on the agents involved (Mol 2002). Although through the use of interviews the views and experiences of individuals do play a role in this paper, the main focus is still on the layered practices in which these actors play a role. This focus is also reflected in the main angle of the interviews: the activities the interviewees are involved in, rather than their views.

Using the concept of ‘enactment’ helps me to take a critical stance toward the practices I studied without treading into the discussion of the technical or biological validity of research results. It enabled me not to treat reality as a single and coherent phenomenon out there that can be reflected correctly or incorrectly in (scientific) representations, but as part of specific practices, that can thus also be arranged differently. That is not to say that questions of technical and biological validity are not important in discussing issues of population identities, but these are not the questions that I aim to address in this paper.

Using the concept of enactment also enables me to look at the connections between population identities as enacted in bioinformatics and genomics, and enactments of population identities in different practices. While these enactments are necessarily different since they are an integral part of the practices in which they occur, they are still connected in different ways as part of a phenomenon (Mol 2002).

Naturalisation, as theorised by several authors (Bowker and Star 2000; Epstein 2007; M’charek 2005), is another important concept for my analysis. Naturalisation occurs when categories and categorised objects become so utterly familiar that they are seen as natural state. Naturalisation thus means that the situated nature of an object or category as local, contingent and created gradually fades and is no longer questioned. As a result the object or category becomes self-evident (Bowker and Star 2000; M’charek 2005).

Naturalisation is often connected with similar processes such as biologisation and essentialisation of a category. Biologisation occurs when a category is seen as grounded in biology, essentialisation when categorical differences are seen as an essential feature of individuals and populations, rather than as resulting from standards that are part of the categorisation practices. As M’charek has argued, naturalisation often leads to essentialisation (M’charek 2005).

In this paper, I mainly use M’charek’s analysis of naturalisation in population genetics. She has convincingly argued that specific technologies and practices in genetics are highly normative, notably when it comes to defining what should count as differences (M’charek 2005). It is from this perspective that I have analysed both cases, studying how values of population identities are materialised in bioinformatics practices. The normative content of technologies and infrastructure tends to be obscured by standardisation: technologies and infrastructure seem to be taken for granted and thus become naturalised, often to the extent that even the appearance that they are resulting from work, let alone that they embody values, is obscured (M’charek 2005; Bowker and Star 2000). This effect is even stronger when a technology is used in different fields of study, moving certain narratives from one field to another. Lipphardt and Niewöhner, in a study on diversity and standardisation, have shown how stories of (evolutionary) origin, or ‘biohistorical narratives’ as they call them, are shaped on the basis of a few concepts like mutation, selection and drift. These narratives move from evolutionary biology to other fields, notably biomedicine. The new field does not have the conceptual framework to lay bare the implicit assumptions of those narratives, so that they become easily naturalised (Lipphardt and Niewöhner 2007).

## Case study one: naturalisation through software tools

### Grouping populations ‘objectively’

*Structure* is a software program that was developed by Prichard et al. to ‘infer population structure and to assign individuals to populations’ based on genotype data (Pritchard et al. 2000). In effect, the program assigns individuals to populations based on genetic markers on multiple loci on the genome. In order for the algorithm to achieve this, certain assumptions have to be made about the data and populations. Some important assumptions are listed here.

- The program can be applied to different types of markers, observing the assumption that the loci of the markers are unlinked and at linkage equilibrium within the population. In other words, it is assumed that the markers are not linked (as might happen for example with markers that are sequentially close to each other or that are functionally linked) and that they are thus totally independent from each other for the frequency with which they occur (Pritchard et al. 2000).
- Hardy-Weinberg equilibrium is assumed within populations (Pritchard et al. 2000). This means that, for the sake of argument, the model assumes the idealised state of random mating within a population.
- A model is assumed in which there are K populations, with the possibility of K being unknown and thus inferred from the data (Pritchard et al. 2000).
- Both the assumption that there is admixture and the possibility that there is no admixture between populations are possible settings in the software (Pritchard et al. 2000).
- Prior knowledge of sampling location (or other characteristics of individuals) can either be used to improve accuracy at assigning individuals to populations, or can be left out to improve validity. In the case of using the information, it is assumed that sampling location is usually connected to population membership, detecting immigrants through that assumption. It is suggested however, that data should at first be clustered *without* using geographic information to check if genetic and geographic clusters overlap (Pritchard et al. 2000).

These assumptions are the choices forming the base for the algorithm. For the algorithm to work, the ‘reality’ it ‘represents’ needs to be subjected to certain assumptions that serve to reconcile mathematical and material reality. This means that not only mathematical considerations find their way into the algorithm through these assumptions, but also included is the way the authors perceive the material reality that they are trying to model.

In the paper describing the algorithm, the authors claimed that the definition of populations is typically ‘subjective’, based, for example, on linguistic, cultural or physical characteristics (Pritchard et al. 2000). They saw this as a problem for population genetics, which they addressed by building *Structure*:

‘[I]t may be difficult to know whether a given assignment of individuals to populations based on these subjective criteria represents a natural assignment in genetic terms, and it would be useful to be able to confirm that subjective classifications are consistent with genetic information and hence appropriate for studying the questions of interest.’ (Pritchard et al. 2000)

The quote shows some of the reasons for creating the software as well as underlying perceptions of reality. Both these reasons and these perceptions find their way into the design of the program.

The designer choices make *Structure* suitable for certain kinds of research. For example, making it possible not to use any prior knowledge of the origin of data in ‘subjective’ populations makes it a good tool for comparing genetic population structures to what the authors call subjectively defined populations. The possibilities of the program thus guide the use of the software in the direction of what the makers had in mind when designing it, even though it can be used for other purposes as well. This guiding effect is strengthened when the users of the program read the paper, because the purpose is clearly stated there.

Underlying perceptions of reality also find their way directly into the algorithm. This is most clearly the case in the choice for the basic model of K populations. The assumption of K populations connects the model to perceptions of what the authors call ‘subjective’ defined populations, based on linguistic, cultural or physical characteristics in the sense that it presupposes clusters of individuals that are part of the same identity. The use of the term ‘subjective’ suggests that genomics sequences contain *objective* or even *natural* criteria for defining populations, which can be accessed through the use of *Structure*. By connecting subjectively defined populations to genetic clusters, they become anchored in the genes, biologising those populations. Allowing for a model without admixture as well as one with admixture, carries the assumption of discrete populations as origin. Admixture assumes that some individuals originate from multiple populations (Pritchard et al. 2000), thus positioning discrete populations more in the past, as an origin that might have become mixed since, but to which individuals still do or do not belong.

To see how the assumptions underlying the *Structure* algorithm might work out in the use of the program in research practices, I will focus on one particular study by (Rosenberg, et al. 2002a) that was performed with the help of *Structure*. The reason for choosing this specific study is that it aims at clustering human population structures as ‘naturally’ as possible, comparing this ‘natural’ clustering with geographical location, thus using the program for a study as intended by its makers. Moreover, as it is presented as an example study on the website of these makers (Pritchard 2000) and co-authored by one of them, it seems an adequate illustration of what kind of study the programmers had in mind when designing this software.

In the study, a dataset (cell line panel) from the Human Genome Diversity Project (HGDP) was used, containing so called ‘microsatellite loci’ (short stretches of highly variable DNA) from several indigenous populations^3^ all over the world. An indigenous population in the context of the HGDP is a group of individuals from a certain geographical location, whose ancestors are supposed to have lived in that same location for many generations. There is a preference for populations living as isolated from other populations as possible. In the panel these populations are referred to in terms of ethnicity or nationality.

In their study, Rosenberg et al. used *Structure* to divide the datasets into a given number of clusters (K) with genetic difference as the only criterion. At K = 2 up to K = 5 the clustering shows a clear resemblance to major geographical regions with natural barriers such as oceans and high mountains. At K = 2 Africa, Europe, the Middle East and Central/South Asia were clustered together; within the other cluster was East Asia, Oceania and America. Each successive clustering, increasing K with one cluster, split one of the previous clusters in two. An ‘unambiguous’ set of clusters was still found at K = 6 but here an isolated single population, not resembling a major geographical region split off. Further clustering of the entire dataset resulted in multiple clustering solutions (Rosenberg et al. 2002b).

In short, the results seem to indicate that large geographical regions are reflected in the genome. Although not presented in such terms, this division is nonetheless reminiscent of divisions in terms of race, a form of essentialisation that has been vehemently problematised, both in scientific and in societal discourse. Both the specific *use* of the software, such as the choice for a panel from the HGDP, and the software itself are constitutive in the enactment of populations. The assumptions and decisions embodied in the algorithm, such the assumption of clustered populations and admixture, are essential in the enactment of populations resulting from the study. In this way ‘subjective’ categories become enacted as biological categories, lending the concept ‘population’ as well as the specific divisions presented in the research, a kind of naturalness or objectiveness that the subjective categorisation lacks.

Genetic criteria are thus matched to subjective population structures, thus serving as objectifying confirmation of structures that were based on subjective criteria, biologising those existing definitions.

In a paper on the Rosenberg study, partly based on ‘personal communication’ with Rosenberg, Bolnick concluded that the mention of either 5 or 6 genetic clusters in nearly all references to the study by Rosenberg et al., results from these numbers fitting ‘the general notion in our society that continental groupings are biologically siginficant’ (Bolnick 2008). She thus suggested that readers, rather than the authors, make these connections. This ‘genral notion’, however, is very present in the way Rosenberg et al. presented their results, for example when they emphasise the six clusters and their connection to geographical regions in the abstract of their paper (Rosenberg et al. 2002a) or in their suggestion of what the results of their study can be used for:

‘General agreement of genetic and predefined populations suggests that self-reported ancestry can facilitate assessments of epidemiological risks but does not obviate the need to use genetic information in genetic association studies.’ (Rosenberg et al. 2002a)

This link to predefined populations and self-reported ancestry, even if not absolute, confirms a natural base for these categories *and* links them to the biology of individuals. This is in line with the broader notion that was introduced by Prichard et al. as one of the main reasons for writing the program: that linguistic, cultural or physical characteristics might correspond with genetic information. In the next section I will focus on the role of computers in enacting this connection.

### Computers as naturalising technology

Bioinformatics as a scientific discipline, as well as some of its tools, such as ready-to-use software packages for biologists, have the tendency to blend into the background of the research for those regarding them as infrastructure. As a result, parts of the practices of bioinformatics remain largely invisible. One bioinformatics researcher referred, in an interview, to this invisibility of the discipline in the context of grant proposals. What is often missing or underestimated in research proposals, are the costs for the bioinformatics part of research, because it is seen as part of the infrastructure of the research institution. Interestingly, this topic came up in the context of the invisibility of the work that goes into the development of software tools. As with any other software that is developed for use by non-software experts, the success of these tools in bioinformatics partly depends on their ease of use. As this interviewee expressed it, ‘for biologists there always has to be a button with some visible elegance and for the rest, not too much hassle.’ This opacity reflects Bowker and Star’s observation that infrastructures tend to become invisible when they are working well, obscuring the choices that are made (Bowker and Star 2000). Thus, the work and costs of bioinformatics, together with the values this work entails, submerge into the background when bioinformatics comes to be regarded as infrastructure. As a result, the constitutive role of bioinformatics in the design of research practices and research results is often overlooked, adding to their naturalisation.

In an interview with a population geneticist, he referred to the ease of use of *Structure* and how that may cause incorrect or unreflexive use of the program. He worded it as follows:

‘Look, the program *Structure* is in principle a model-free program. So it tests data without you attaching a label to that data beforehand. But you can manipulate the data tremendously. Only, the problem is, most people manipulate it without being aware that they are manipulating it. I see that as the dilemma using this kind of program. It can be horribly abused this way. But because the pictures are so pretty and they are so simple. (…) It has such an impact, because it says more than a table with numbers. So it is wildly popular.’

So the attractively visualised output leads to less attention as to what the program really *does* to the data. Moreover, as the same population geneticist pointed out in the interview, since samples used with *Structure* are usually aimed at selecting for *difference*, conclusions that are drawn about genetic differences between populations nearly always contain overstatements about these relations. In the Rosenberg study, the choice for a panel with indigenous populations is already a choice for clustered populations. The outcome of their study is a clustering of already clustered individuals, rather than of a random sample, thus overstating the continental clustering. And while Rosenberg et al. were undoubtedly well aware of the technical details of the program, the seemingly simple visualisations helps the *reader* of their study to read the outcomes of the software without much knowledge of how the software works. The visualisations can thus add to easy acceptance of the connections between existing populations and newly created genetic categories as established by the software.

What the population geneticist quoted above refers to when calling *Structure* a model-free program is that *Structure* can infer population structures from genetic data without predefining populations based on non-genetic data. It does not mean that there is no model underlying the software. This lack of pre-definition positions the software as a more or less unbiased mediator, giving the impression that the data can speak for themselves, conveying their meaning through the software with a minimum of biased mediation by human interpretations.

I encountered this idea of the computer or its software as an unbiased mediator in different contexts during my research, under a variety of names such as ‘unbiased grouping’ or ‘the data speaking for themselves’. One of the bioinformatics experts I interviewed, addressing the diversity of the concept ‘function’, expressed it as follows:

‘The only thing that could still be interesting, is of course to what extent this kind of thing can all be automated… the whole scientific process. So induction and hypothesis testing, because you would prefer the data to make its own definition of function… (…) a natural definition, a natural ontology. So that it does not come from our interpretation of how a cell functions but from how the data itself thinks it looks like.’

While this is a description of an ideal rather than of an existing reality, it implies that if the scientific process *were* to be fully automated, the data could *speak for themselves*. In other words, automation is the process of rendering the results more natural, with the computer as an unbiased neutral agent, reading nature directly, without conceptual mediations. This could strengthen essentialisation, because the outcomes tend to be regarded as facts from nature, rather than as human created and thus contestable narratives.

What we see here, then, is the naturalisation of population structures through the naturalisation of technology, as M’charek described it (M’charek 2005). The computer, software and algorithm are seen as tools that allow the data to speak for themselves. The software thus takes the place that used to be taken by the scientist, the place of the modest witness ‘whose accounts mirror reality’ (Haraway 1997). This creates a situation where the context-dependent values that are embedded in this software and the practices that it is part of are no longer recognised, resulting in the naturalisation and often essentialisation of, in this case, enactments of populations.

### Discussion

This case study shows how bioinformatics software can contribute to specific enactments of population identities through assumptions that become embedded in an algorithm. In these enactments, populations as biological and genetic phenomena are naturalised, resulting in the essentialisation of differences between populations. These essentialised biological differences reconnect with notions of ethnicity and race.

Fujimura and Rajagopalan have suggested a way of avoiding essentialisation of identity in the context of genomics. Focussing on circumventing the use of racial categorisations in biomedicine, they argue for a rigorous separation of existing population identities and genetic clustering, by creating new ‘categories of genetic similarities’ for biomedical purposes. They took this idea from their ethnographic fieldwork on bioinformatics in biomedical practices, where some researchers are already arguing for and implementing such an approach. In this approach, in which the possibilities of bioinformatics play a central role, new categories should be based on genetic data only and leave out information on shared ancestry, race and ethnicity (Fujimura and Ramya 2011). This way, the essentialisation of existing identities would be prevented.

Ignoring existing categories is thus technically feasible, using data-driven categorisations like with *Structure*, but with the intent to get *away* from what Prichard et al. have called subjective definitions of populations. However, even in those instances where new and allegedly more neutral population categories are created, connections to existing ethnic and racial categories tend to slip back into the discourse, as Fujimura and Ramya themselves have observed (2011). Lessons from the *Structure* case suggest that whenever this happens, the new population categories that are meant to be out of reach of the ‘cultural bias’ of existing population identities, can reconfirm those identities as biologically grounded, thus strengthening possible essentialisations in the same way that it happens in the case of *Structure*.

Thus, a priori presuppositions about populations tend to find their way back into genetic narratives. The assumed neutrality of the computer cannot prevent this and, as an assumption, can even reconfirm and strengthen these presuppositions in certain practices. I will come back to this in the conclusion. The next case study shows how genetics and bioinformatics not only play a role in reconfirming existing presuppositions about populations, but can also play a role in biologising national identity, an aspect of population identity that is not generally seen as biological.

## Case study two: reference sequences: normal genomes, normal bodies

### Capturing genetic nationality in a reference database

The Genome of the Netherlands is a large-scale genome-sequencing project, carried out by a consortium of five university medical centres in the Netherlands. Because this project involves processing large amounts of data, bioinformatics is not a background infrastructure but one of the main disciplines involved. However, since the goal of the project is to create a *reference* database, it is likely that the product of the project will become part of the infrastructure of genomics research in the Netherlands, naturalising certain values embedded in the database.

The aim of the project, according to the project’s website, is to map the genetic variation in what they call the ‘Dutch indigenous population’ (Genome of the Netherlands 2011). As Wijmenga, the director of the project, explained in a published interview, the mapping is done by sequencing the genomes of 750 samples from Dutch biobanks, consisting of samples from 250 parent couples and one child for each couple, ‘representing healthy people living in all the different regions of the Netherlands (equal numbers from all the original 11 provinces^4^ and a few extra from Amsterdam and Rotterdam)’ (van Megchelen 2010).

Wijmenga explained that, while comparing the results of the project internally could yield interesting results, for example showing regional genetic differences, the main aim of the project is the building of a reference database for future use in biomedicine. To this end the data from the sequenced samples is used to identify local genetic variants involved in health and disease in existing data from Genome Wide Assosation Studies (GWAS) in the Netherlands. This is done using imputation (van Megchelen 2010), a method for statistically determining bits of unknown sequence in-between known variations. Based on this combined information, van Megchelen suggests, DNA chips could be made for these local variants so they can be traced in individuals (van Megchelen 2010).

The main genetic reference for humans is largely based on the sequence created by the Human Genome Project. As one bioinformatics researcher told me:

‘We still depend on the so-called “reference genome” you see. (…) That is made up out of three different people whose genome has been determined and that is now used as the “normal human”.’

Question: ‘is that still the genome of the Human Genome Project?’

‘There have been some updates but it is still based on it. And there are different versions (…). There is for example one specific part on chromosome 17, which is very often different in the European population from the rest of the world. And that, for example, is now an alternative bit, added for that region. (…) So these things are increasingly added and are put as much as possible in one thing, in one form. So that is what we have to compare it with every time.’

Thus, already in the Human Genome itself some population differentiation is taking place, based on geographical regions. Apparently, one reference genome for defining all humans genetically is deemed by researchers to be not always specific enough for research purposes. One bioinformatics researcher, himself involved in the Genome of the Netherlands project, phrased the need for differentiation as follows:

‘[B]efore, those populations did not seem to be so far apart but now that they have started to measure much deeper^5^, you can find variants that are very rare in populations, less than five percent for example. And if you then make a Venn-diagram^6^ of, say, Africans and Europeans and Asians, you see that they are pulling apart, because there are simply so many unique variants.’

The rationale behind the creation of more differentiated reference genomes that select people into genetic proximate clusters, is that with a higher genetic proximity of reference genomes to research subjects, more meaningful differences can be found. Using existing population categories as selection criteria is one way of creating differentiation, a way that is used in many genome projects. A well known example is the 1000 Genome project, an effort to sequence a thousand complete genomes from (mostly indigenous) populations all over the world (1000 Genomes 2008).

In the Genome of the Netherlands project, the differentiation criterion for setting up a genetic reference database is nationality. To be clear, in genetics, nationality is not usually seen as being a priori present in the genes. When the British Border Agency tried to use genetic tests for helping to infer nationality of refugees, *Nature* published an editorial stating ‘the idea that genetic variability follows man-made national boundaries is absurd’ (*Nature*^2009^).

So under certain circumstances the idea of genetic nationality is out of the question, while in the context of reference genomes like the Genome of the Netherlands, genes and nationality seem to go together quite well. The same researcher that is quoted above, when asked about the sample selection, said:

‘Well, what we did is that we took only, so to speak, Caucasian Dutch, otherwise it would be very complicated. And for the rest we did not ask too much, except that their parents were born in the Netherlands. That is more for practical reasons because as soon as you start stratifying, it gets complicated very fast. So we just said, this is more or less the Dutch and if we measure seven hundred and fifty, it will work out in that we will at least find the variants that are specific for Dutch.’

In the context of creating databases containing more proximate reference genomes for biomedical research, it seems sensible to somehow categorise humans. Asked about sampling, this researcher made clear that for *practical reasons* the categories race and ancestry are used as *general criteria* for selecting an indigenous Dutch population.

On the website for the project, which is bilingual, the Dutch word *autochtoon* is used, where the English version of the site mentions ‘indigenous’ (Genome of the Netherlands 2011). This choice of words is significant in light of the criteria used for selecting a Dutch population. The word *autochtoon* (from the Greek, meaning the same land/soil) is paired in the Dutch context with its opposite *allochtoon* (from the Greek for land/soil, ‘foreign’ in English). Both terms are widely used in Dutch political and public debates on issues like migration, asylum policies and crime. The Dutch statistical bureau (CBS) has defined them as follows: ‘For *autochtonen* both parents were born in the Netherlands, while *allochtonen* have at least one parent that was not born in the Netherlands.’ For *allochtonen* born in the Netherlands (2nd generation) the mother’s country of birth counts as country of origin, unless she was born in the Netherlands, in which case it is the father’s country of birth. Subsequently, there is a division between western (Europe, North-America and Oceania, except Turkey) and non-western (Africa, Latin-America and Asia, except Japan and Indonesia) *allochtonen* (Dagevos et al. 2011). This word pair can be seen as a statistical tool that is naturalised in the public debate, where it is used in a common-sense manner for distinguishing between non-western immigrants and their children and other inhabitants of the Netherlands. It is however, not naturalised to the extent that it is uncontroversial. On the contrary, a number of advisory bodies, such as The Netherlands Institute for Social Research (CPB), have strongly recommended abandoning its use. They opt for alternatives such as ‘non-western migrants’ or ‘new Dutch’ (Dagevos et al. 2011). Thus, the word pair *autochtoon* and *allochtoon* distinguishes between people ‘from here’ and people ‘not from here’, regardless of whether they are Dutch citizens or were born in the Netherlands. These discourses are played out in the Dutch political arena and the Dutch media with increasing explicitness.

The choice for the word *allochtoon* thus places the Genome of the Netherlands project firmly within the mainstream societal discourses regarding who does and does not belong. But more importantly, this choice of terminology signifies the selection criteria used for Dutchness in the project, importing a specific, problematic, common sense notion of Dutchness as a form of identity into the reference database.

### Bioinformatic connections and stabilisation of identities

Neither the Genome of the Netherlands, nor the Human Genome as such can be found ‘out there’ in nature. As reference databases they are themselves technologies in the practices of genomics: they are created phenomena that result from work and choices, including choices concerning what gets included and what gets excluded. The configuration of the Genome of the Netherlands database, whose parameters are more or less random: parents born in the Netherlands, Caucasian phenotypic features and the availability of certain samples in the participating Dutch biobanks, enacts Dutchness as a genetic category.

M’charek has argued that bodily differences can be fragile or more durable in their enactment. Fragile meaning that they are short-lived and can shift from one moment to another. Durable meaning that they are lasting, often because they are naturalised within practices and technologies (M’charek 2010). The criteria for what counts as Dutch and non-Dutch genetically are stabilised in the materiality of the database in the sense that they are contained in the genomes that are stored there. That is not to say that with the database genetic Dutchness is absolutely fixed in time and space, because reference databases and their connections do change over time, but in the sense that it is made to be more durable.

The stabilisation or durability of genetic Dutchness in a database matters where this database is involved in the enactment of Dutchness. The creation of the Genome of the Netherlands is in itself a way of biologising nationality in the sense that Dutchness is enacted as a genetic category. Here, bioinformatics is involved as a major contributor to the creation and maintenance of databases. More specifically, however, the database is involved in enactment of Dutchness where it is used in its role as a reference. Here it is connected to other databases as well as to individuals. It is in this connection that bioinformatics plays a crucial role, because these connections are partly made *in silico*: in the computer. Points of connection are genetic variants, places where the genome differs between individuals, in this case focussing on differences specific for the Dutch population.

As mentioned before, the 750 genomes from the Genomes of the Netherlands project will be used to enhance earlier GWAS data through imputation. In the GWAS data, variations in the genome on single nucleotides (so called SNPs) are known, but not the nucleotides in between. SNPs from the GWAS data and SNPs in the fully sequenced genomes are matched and subsequently the ‘gaps’ in the GWAS data are inferred statistically from the data in the known genomes. In this process, SNPs on the reference and the GWAS data are matched and only where there is a match, imputation will be possible. The imputation process is thus a process in which the Genome of the Netherlands data, as reference, is connected to existing databases in an attempt to find more local genetic variants.

The next step, as Wijmenga suggested, could be the creation of chips for specific local variants. That means that micro array chips will be created, which can detect the presence of specific variations in the genome of an individual, thus matching the individual to the known variation in the database. Since these chips will focus on genetic local populations, they will specifically serve an indigenous population. It could be argued that it is the selection of individuals to whom the chip is applicable that separates *autochtoon* from *allochtoon*, not the chip itself as technology. However, the specific connections enabled by bioinformatics practices and technology, connecting the chip to the Genome of the Netherlands, create the conditions in which this selection is part of a practice where it can be naturalised and possibly essentialised.

### Discussion

As M’charek has argued, reference databases tend to become naturalised. As a result, the work and choices that go into making them are often not considered in their routine use as a reference. Through the naturalisation of the reference genomes, the objects they help create, such as individual genomes, tend to be naturalised as well (M’charek 2005). It is not unlikely that with the Genome of the Netherlands, the choice for what counts as Dutch and non-Dutch will be repeated through its common sense character. This aspect is already naturalised in the Dutch context. That is, the selection of who is indigenous Dutch and who is not, is based on this common sense notion rather than based on the choices concerning which genomes were included in the Genome of the Netherlands. With the routine use of the Genome of the Netherlands as reference database, Dutchness as a genetic category could become biologised along the lines of *autochoon* and *allochtoon* distinctions. Essential in this biologisation is the connection between database and individual, a connection that is enabled by relating *in silico* sequences to DNA in the cell, a practice in which bioinformatics plays a crucial role. This relation may seem self-evident. After all, the four-letter sequence (ATCG) in the computer or database reflects the order of nucleic acids in the cellular DNA. However, rendering the order of nucleic acids computer-readable as an *in silico* sequence or four letters involves technology and human work. In the process, the order of nucleic acids in the cell is enacted as a base for the computation of genetic functions and genetic connections. The meaning that is given to sequences in this process is naturalised by the seemingly self-evidence of this relation. With the advent of Next generation sequencing, the work is increasingly automated in an informatics process running in the background from the point of view of biologists and thus getting out of view from their perspective, further naturalising the meaning of connections between *in silico* sequences and living bodies.

## Conclusion: bioinformatics and population identity

In this article, I have tried to tease out the ways in which bioinformatics, as a discipline, is involved in the enactment of population identities. I presented two cases of bioinformatics technologies that play a role in such enactments: the software program *Structure* and the reference database Genome of the Netherlands. In both cases, information technology and bioinformatics as a discipline play a role in the enactment of population identities in a different manner.

In the *Structure* software, a priori presuppositions about populations find their way into the algorithm, such as the assumption of clustered populations as well as a sense of origin, be it mixed or not. The purpose of the software is to see if categorisations according to so called ‘subjective’ criteria such as linguistic, cultural or physical characteristics agree with genetic clusters. While this purpose is not directly built into the algorithm, it is reflected in the possibilities of the software as well as in the article that accompanies the software. These aspects of algorithm, software and the accompanying narrative, are essential ingredients for an enactment of a division of populations according to continental barriers in the Rosenberg study, that are reminiscent of racial divisions. This is not to say that the way the study is conducted does not matter for the outcome, the selected data for the study in the form of an HGDP panel is an important element too. I am rather arguing that bioinformatics plays a significant *part* in this enactment and certainly not a neutral one.

Showing that bioinformatics is not neutral in these enactments is important. Bioinformatics and computers are not only relevant because they can embody certain assumptions, but they also play a role in the naturalisation of connections between genetic and ‘subjective’ populations. While acknowledged as an important enabler of life science research, bioinformatics is regularly regarded as infrastructure for this research. As a result, its contributions and the values contained in them get easily overlooked as *constituting* part of the research and the values contained in this research. The supposed neutrality of computers within genomics can create the idea that data speak for themselves, that through them nature can be perceived without inevitable human bias, black boxing and naturalising implicit assumptions that are part of not only the tools but also of the broader discourses surrounding genetics. These naturalisations can easily lead to essentialisations of differences that are located in the bodies of people who can be identified as a group in everyday life.

While the Structure case thus shows the role of bioinformatics in the re-enactment of biological differences in racial and ethnic categories as genetic differences, the Genome of the Netherlands shows the role of bioinformatics in the biologisation of a different origin narrative: that of nationality. Here, it is the database itself that, through its contents of 750 sequenced genomes, enacts Dutchness as a genetic category by representing the genomic variation within the Dutch indigenous population. This could be regarded as a purely statistical representation. However, the connection of individual genomes to, in many ways, a naturalised common sense definition of indigenous Dutchness, is more than ‘just’ statistical. Through these connections the database materially connects this naturalised definition to individual genomes and thus bodies, enacting (Dutch) nationality as a genetic category. It is in these connections that bioinformatics plays a crucial role.

Next to being a constituting factor in the enactment of genetic Dutchness, as a reference, the database also stabilises this enactment. Embodying genetic Dutchness as represented by 750 full genomes and identified local variants, its repeated use as a reference database can further stabilise this enactment.

In the case studies that I presented in this paper, bioinformatics is not the only constituting factor in the enactments of population identities as biological. What I wanted to show is how bioinformatics is an essential element in these enactments and how its tools and practices do not only enable the research, but also contribute to enactments that are related to research results, be it by the embodiment of certain enactments in algorithms and databases or through the naturalising effect of supposed neutrality of computers, bioinformatics is not a neutral element in the enactment of population identities as biological.

Bioinformatics as a research practice is an important player in genomics as well as other fields within the life sciences. Not many projects in genomics can do without its support. Acknowledgment of the work of bioinformatics as a research field that has an important influence on how research is done in the life sciences, should go together with the acknowledgment of the ways in which bioinformatics, far from being neutral, entails values and specific enactments of specific human identities.

### Flexible identities

‘To classify is human’ as Bowker and Star have written in their introduction to *Sorting things out* (Bowker and Star 2000). As humans, we are standardising and categorising continuously, from sorting the dishes up to formal bureaucratic systems of classification that allow for the organisation of large systems like medical care. Dividing people into various kinds of groups is part of this classification effort. As humans we engage in this more or less continuously both in our everyday lives and in more formal settings. It is part of what we do to make sense of the world. The two cases here presented show different ways in which bioinformatics is involved in the enactment of population identities, notably through classification or categorisation work. Given that (human) classification is part of human existence, the genetic categorisation of people in a research context is not self-evidently problematic. As Epstein as well as Rose have pointed out, in the contemporary power relations, racial and ethnic classifications are often used as a means to claim equal rights (Epstein 2007; Rose 2007). Where these classifications are biologised and essentialised however, they are no longer flexible categories that one can choose to adopt as part of one’s identity. Genetics is certainly not the only practice in which essentialisation takes place. But as a practice where new biological truths seem to be found, it can certainly add a strong voice to biologisation.

In the discussion of the *Structure* case, I mentioned the suggestion by Fujimura and Rajagopalan to avoid biologisations and essentialisations of identity in the context of genomics by creating new categories based only on the genome (Fujimura and Ramya 2011). Apart from the fact that practices seem to resist this option, the argument for separation of genetic structures from existing population structures seems to entail a relapse into the idea that nature and culture are separable, as well as the idea that scientific categories can be neutral vis-à-vis the cultural and political, this time aided by computers. Such a separation would also make it more difficult for social sciences and philosophy to talk about the biological. In other words, it might strengthen the hegemony of the natural sciences over knowledge about the biological and the body rather than ‘neutralising’ it. As Mol has argued, building on Haraway, separating the social realm from the biological in research about the body impoverishes knowledge about the body because it causes certain realms to be self-evidently natural, depoliticising aspects of the body that are inherently political (Mol 2002).

In short, while the solution that Fujimura and Rajagopalan have offered could counter essentialisation of population identities to a certain extent, in the long run it can have the adverse effect of strengthening essentialisation. I would therefore like to suggest a different type of solution. In view of the ubiquity of genetics in many research disciplines, with bioinformatics as its infrastructure, genes are bound to play a role in identity work. In this light I think the key question is to what extent population identities could be kept flexible, without separating nature from culture and facts from values. Below I sketch possibilities for keeping identities flexible in the two cases here studied. These should be regarded not as formal recommendations, but as general suggestions for alternative ways of thinking.

Keeping identities flexible in a software program like *Structure* could mean considering whether it is possible to diversify the stories of origin implicated in the program, showing the complexities of actual origins. This could be done by emphasising the gradual rather than discrete genetic differences between people in a) the documentation accompanying a program like *Structure*, b) its graphics and c) the studies it is used for. Tishkoff and Kidd for example, have shown how such a gradual perspective can lead to a diversified view in which complex individual ancestry can play a role but in which a crude division by race or a simplified use of ethnicity does not make sense (Tishkoff and Kidd 2004).

In the case of the Genome of the Netherlands, keeping identity flexible could mean considering to what extent a Dutch reference database would be feasible that somehow reflects the genetic diversity of the entire current Dutch population, without losing its effectiveness as a reference. The focus on the uniqueness of the indigenous or *autochtone* Dutch population(s) is based on common sense ideas of origin and belonging that are repeated through this focus. If the focus would be on genetic sameness and difference within the *entire* current Dutch population, that would mean there is more room for contingency and surprise, rather than of staying within the boundaries of the expected. Some variants might show some connection to a diverse ancestry, while other variants might overlap or follow other patterns. This could aid the diversification of stories of origin for different ethnic groups, while possibly fragmenting individual stories of origin, thus creating flexibility.

With this kind of adaptation, the connection between identities as they are enacted through bioinformatics tools and actual living individuals could interfere with other enactments of population identity in interesting ways, enlarging the potential for diversification, thus countering rather than furthering essentialisation.

## Endnotes

^1^The concept ‘enactment’ is elaborated on in the ‘Methods and concepts’ section.

^2^All interviews were conducted in Dutch; quotes are translated by the author.

^3^Rosenberg et al. did not explicitly mention the indigenous origin of the samples in their article, nor was it mentioned in the original publication of the panel (Cann et al. 2002). This does not mean that the origin of the samples is hidden, as the Diversity Project’s goal is to gather genetic information from indigenous populations worldwide. Rather, it shows how self-evident the use of samples from indigenous populations is for these researchers.

^4^The Netherlands has consisted of 11 provinces between 1840 and 1986, when Flevoland was added by way of a land re-claim. The reference to the *original* 11 provinces presumably means that Flevoland was not included in the sample.

^5^Measuring deeper means that larger sequences are used for the research.

^6^A Venn diagram shows the relationship between sets. African, European and Asian pulling apart thus means that these ‘sets’ are mutually divergent on the basis of genetic variance.

## Authors’ information

Jan van Baren-Nawrocka obtained his master degree at the University for Humanistics in Utrecht, the Netherlands. Currently he works as a PhD researcher on a CSG project on Bioinformation and human identity at the Radboud University Nijmegen, the Netherlands.

## References

[CR1] 1000 Genomes (2008). 1000 Genomes: a deep catalog of human genetic variation.

[CR2] Bolnick DA, Koenig BA, Lee SS-J, Richardson SS (2008). Revisiting race in a genomic age. Individual ancestry inference and the reification of race as a biological phenomenon.

[CR3] Bowker GC, Star SL (2000). Sorting things out: classification and its consequences: 1st MIT Press paperback Aufl: inside technology.

[CR4] Cann HM, De Toma C, Cazes L, Legrand M-F, Morel V, Piouffre L, Bodmer J (2002). A human genome diversity cell line panel. Science.

[CR5] Dagevos J, Mérove G, Willem H (2011). Jaarrapport integratie 2011.

[CR6] Epstein S (2007). Inclusion: the politics of difference in medical research: Chicago studies in practices of meaning.

[CR7] Fujimura JH, Ramya R (2011). Different differences: the use of ‘genetic ancestry’ versus race in biomedical human genetic research. Social Studies of Science.

[CR8] Genome of the Netherlands (2011). Ultra-sharp genetic group portrait of the Dutch.

[CR9] Haraway DJ (1997). Modest_witness@second_millennium femaleMan©_meets_OncoMouse™: feminism and technoscience.

[CR10] Lipphardt V, Niewöhner J (2007). Producing difference in an age of biosociality: biohistorical narratives, standardisation and resistance as translations. Science, Technology & Innovation Studies.

[CR11] M’charek A (2005). The human genome diversity project: an ethnography of scientific practice: Cambridge studies in society and the life sciences.

[CR12] M’charek A (2010). Fragile differences, relational effects: stories about the materiality of race and sex. European Journal of Womens Studies.

[CR13] Van Megchelen P (2010). Unique in-depth perspective on regional genetic variants. Hub: Neweletter of the Biobanking and Biomolecular Resources Research Infrastructure Netherlands (BBMRI-NL).

[CR14] Mol A (2002). The body multiple: ontology in medical practice: science and cultural theory.

[CR15] Nature (2009). Genetics without borders. Nature.

[CR16] Pritchard JK (2000). Structure.

[CR17] Pritchard JK, Stephens M, Donnelly P (2000). Inference of population structure using multilocus genotype data. Genetics.

[CR18] Rose N (2007). The politics of life itself: biomedicine, power, and subjectivity in the twenty-first century: information series.

[CR19] Rosenberg NA, Pritchard JK, Weber JL, Cann HM, Kidd KK, Zhivotovsky LA, Feldman MW (2002). Genetic structure of human populations. Science.

[CR20] Rosenberg NA, Pritchard JK, Weber JL, Cann HM, Kidd KK, Zhivotovsky LA, Feldman MW (2002). Supporting online material for ‘genetic structure of human populations’. Science.

[CR21] Tishkoff SA, Kidd KK (2004). Implications of biogeography of human populations for ‘race’ and medicine. Nature Genetics.

[CR22] Zwart H (2009). Genomics and identity: the bioinformatisation of human life. Medicine Health Care and Philosophy.

